# Revisiting Meiosis in Sugarcane: Chromosomal Irregularities and the Prevalence of Bivalent Configurations

**DOI:** 10.3389/fgene.2018.00213

**Published:** 2018-06-14

**Authors:** Maria Lucia C. Vieira, Carmelice B. Almeida, Carlos A. Oliveira, Luana O. Tacuatiá, Carla F. Munhoz, Luiz A. Cauz-Santos, Luciana R. Pinto, Claudia B. Monteiro-Vitorello, Mauro A. Xavier, Eliana R. Forni-Martins

**Affiliations:** ^1^Escola Superior de Agricultura “Luiz de Queiroz”, Universidade de São Paulo, Piracicaba, Brazil; ^2^Instituto de Biologia, Universidade Estadual de Campinas, Campinas, Brazil; ^3^Centro de Cana, Instituto Agronômico de Campinas, Ribeirão Preto, Brazil

**Keywords:** *Saccharum* spp., meiotic behavior, meiotic irregularities, FISH, centromeric probes, chromosome associations

## Abstract

Traditional sugarcane cultivars (*Saccharum officinarum*) proved highly susceptible to diseases, and this led breeders to progress to interspecific crosses resulting in disease resistance. A backcrossing program to *S. officinarum* was then required to boost sucrose content. Clonal selection across generations and incorporation of other germplasm into cultivated backgrounds established the (narrow) genetic base of modern cultivars (*Saccharum* spp.), which have a man-made genome. The genome complexity has inspired several molecular studies that have elucidated aspects of sugarcane genome constitution, architecture, and cytogenetics. However, there is a critical shortage of information on chromosome behavior throughout meiosis in modern cultivars. In this study, we examined the microsporogenesis of a contemporary variety, providing a detailed analysis of the meiotic process and chromosome association at diakinesis, using FISH with centromeric probes. Chromosomal abnormalities were documented by examining high quality preparations of pollen mother cells (700 in total). Approximately 70% of the cells showed abnormalities, such as metaphase chromosomes not lined up at the plate, lagging chromosomes and chromosomal bridges, and tetrad cells with micronuclei. Some dyads with asynchronous behavior were also observed. Due to the hybrid composition of the sugarcane genome, we suggest that bivalent incomplete pairing may occur in the first prophase leading to univalency. The presence of rod bivalents showing the lagging tendency is consistent with a reduction in chiasma frequency. Finally, the presence of chromatin bridges indicates the indirect occurrence of chromosomal inversions, although chromosome fragments were not clearly recognized. Possible reasons for such meiotic abnormalities and the large prevalence of bivalent formation are discussed.

## Introduction

Sugarcane is an ancient crop. The early canes were domesticated around 7,000 BCE and the simplest scenario is that *Saccharum officinarum* was domesticated from *Saccharum robustum* in the New Guinea region. Humans then spread these cultigens over large distances. In Southeast Asia, *S. officinarum* hybridized with local *Saccharum spontaneum* giving rise to Indian and Chinese cultivars (reviewed in Grivet et al., 2004). Cultivation and sugar processing had become established in Persia by the 600 CE and within a century had reached the Mediterranean and North Africa. The Spanish and Portuguese conquistadors carried sugar to the southwest of Iberia (Madeira, Canary Islands) and brought sugarcane to the New World.

Despite this long ongoing period of cultivation, the early stages of plant selection occurred fairly recently. From the 6th to the 18th century, all cane grown in the Western world was of a single variety, “Creole” cane from Java. Subsequently, other canes dominated their adopted habitats for lengthy periods in Hawaii, Mauritius (from Java), Australia (from New Guinea), South Africa, and Brazil. However, traditional cultivars proved highly susceptible to diseases, and this led breeders to focus on the hybridization of *S. officinarum* clones in the early 1900s, but they soon progressed to interspecific crosses incorporating the wild *S. spontaneum* resulting in disease resistance. A backcrossing program to *S. officinarum*, called “nobilization”, was then required to boost sucrose content (reviewed in Dillon et al., 2007). Clonal selection across generations and incorporation of other germplasm into cultivated backgrounds established the (narrow) genetic base of modern cultivars, which have a man-made genome (Grivet et al., 2004; Jannoo et al., 2004).

Due to female meiotic restitution, the F_1_ hybrid conserved all *S. officinarum* chromosomes and half the *S. spontaneum* chromosomes (2*n*+*n*), and then a few backcrosses later, this hybrid broke down to *n*+*n*, establishing the constitution of sugarcane (Bremer, 1961a). Current cultivars (*Saccharum* spp.) derived from crosses between varieties or clones, are highly polyploidal and tolerant of aneuploid constitution, which makes the chromosome combination in each offspring unique and unpredictable (Grivet and Arruda, 2002). The crop is grown primarily for sugar production in the warm climates of South America, North America, Asia, and Australia. In recent years, especially in Brazil, sugarcane has become of enormous economic importance with multifaceted end-uses, such as the production of ethanol and bioelectricity (Furlan et al., 2013 and references therein).

Taxonomically speaking, the “*Saccharum* complex” is a closely related group, containing five genera: *Erianthus* (section *Ripidium*), *Miscanthus* (section *Diandra*), *Narenga, Sclerostachya*, and *Saccharum*, encompassing six species forming a wild (*S. spontaneum* and *S. robustum*) and a cultivated group. This cultivated group, in turn, contains *Saccharum edule*, eaten as a vegetable in Pacific islands and Papua New Guinea; *S. officinarum*, the most widely grown species; and *Saccharum barberi* and *Saccharum sinense*. It has been suggested that these last two are interspecific hybrids of *S. spontaneum* and *S. officinarum* rather than true species (D’Hont et al., 2002).

In addition to the breeding techniques that led to the formation of *Saccharum* spp. discussed above, a few natural and resynthesized polyploids are also worthy of consideration. *Brassica napus* is a good example that has been extensively studied. *B. napus* originated from interspecific hybridization between two diploid ancestors, followed by polyploidization. The occurrence of homoeologous exchanges, duplications, and deletions was detected in experimental *B. napus* at the onset of genome merging or within a few generations after the confrontation of the two genomes (see Nicolas et al., 2007; Szadkowski et al., 2010). Moreover, by using fluorescent *in situ* hybridization (FISH) to distinguish all chromosomes present in *B. napus*, Xiong et al. (2011) reported evidence for homoeolog pairing and chromosome rearrangements, aneuploidy, and homoeologous chromosome compensation in the resynthesized plants.

In natural populations as well as in synthetic lines of *Tragopogon mirus* and *Tragopogon miscellus*, two young allopolyploid species (<200 years old), normal bivalent formation and an array of meiotic abnormalities (including multivalent formation, lagging chromosomes, and the formation of anaphase I bridges) have been observed (Lim et al., 2008; Tate et al., 2009). In particular, intergenomic translocations and/or aneuploidy were identified, but variation typically followed a compensated pattern (Chester et al., 2013, 2015). These key reports of such extensive chromosome rearrangements in nature are similar to those observed in experimental *B. napus* allopolyploids (Xiong et al., 2011).

However, the regularity of meiosis needs to be in place soon after polyploid formation and was found to increase rapidly in experimental polyploids that were initially chromosomally unstable (reviewed in Cifuentes et al., 2010). In wheat, for instance, the *Ph1* gene is the major component of a multigenic system inhibiting recombination between homoeologous chromosomes favoring cytological diploidization. Similarly, *B. napus* shows complete diploid-like meiotic behavior, with only bivalents and disomic inheritance; thus, in all euploid genotypes, crossovers mostly occur between homologous chromosomes. The so-called pairing regulator gene *PrBn* is the most influential locus reducing homoeologous pairing in *B. napus* (Jenczewski et al., 2003; Suay et al., 2014). The BYS locus has also been identified as a dominant locus contributing to homologous pairing during meiosis in the allotetraploid *Arabidopsis suecia* (Henry et al., 2014).

The unusual genome complexity and inter-relationships within *Saccharum* (with ploidy levels ranging from 5× to 16×) has inspired several molecular studies that have elucidated aspects of sugarcane genome constitution (Souza et al., 2011; Setta et al., 2014), architecture (Garcia et al., 2013), and cytogenetics, studied mainly by D’Hont et al. (1993; 1996; 1998), D’Hont (2005) and Piperidis et al. (2010). However, there is a critical shortage of information on chromosome behavior throughout meiosis. In this study, we examined the microsporogenesis of the IACSP93-3046 variety, providing a detailed analysis of meiotic process and chromosome association at diakinesis, using FISH with centromeric probes. Possible reasons for meiotic abnormalities and the prevalence of bivalent formation are discussed on the basis of information in the literature and data generated here.

In brief, our hypothesis is that *Saccharum* sp. experienced intergenomic exchanges during meiosis of the interspecific hybrids and nobilized clones. However, during the nobilization process sugarcane acquired the ability to suppress multivalent formation by restricting meiotic crossovers to homologous chromosomes or homologous chromosome regions. We suggest that a genetically controlled strategy that enforces bivalent formation was possibly inherited from *S. officinarum* (an autopolyploid with diploid-like meiotic behavior) and this became evident once the hybrid-derived clones broke down to *n*+*n*, establishing the constitution of sugarcane.

## Materials and Methods

### Plant Material

**FIGURE 1:**
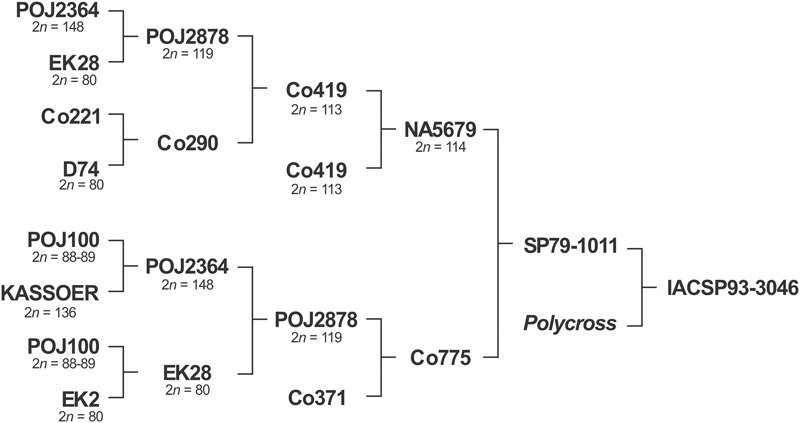
The pedigree of the Brazilian sugarcane variety, IACSP93-3046. Kassoer, a spontaneous hybrid between Javanese *Saccharum officinarum* (*2n* = 80) and *S. spontaneum* (*2n* = 112, the wild Glagah); EK2 and EK28, *S. officinarum* clones cultivated by the natives in Java, Indonesia; D74, a *Saccharum* hybrid; POJ100, *S. officinarum* hybrid, a noble cane; POJ’s, *Saccharum* hybrids obtained on Java Island (robust canes, immune to sereh disease); Co, early cultivars improved in Coimbatore, India. Note that POJ2878 can be found in the pedigrees of almost all the dominant varieties grown worldwide, and many countries with breeding programs of their own continue to rely on Co clones as a germplasm base. NA, varieties bred in Argentina; SP and IAC, in Brazil. Chromosome numbers taken from the literature.

In this study, we investigated commercial sugarcane variety IACSP93-3046 (**Figure 1**), which was released in 2005 and bred at the Agronomic Institute (IAC), a traditional Brazilian public research institute founded in 1887. It exhibits desirable agronomic traits such as good tillering, resistance to sugarcane brown rust, and high levels of sucrose. Due to its erect stool habit, it was recommended for mechanical harvesting. More recently, IACSP93-3046 was the male parent of a full sib progeny used for mapping purposes (Garcia et al., 2013; Costa et al., 2016).

In addition, we used the “Caiana Fita” clone of *Saccharum officinarum* (2*n* = 80) as a control in FISH assays to determine the chromosome-pairing configurations in pollen mother cells (PMCs) of IACSP93-3046 at diakinesis. Stalks and inflorescences were collected at the IAC hybridization station in Bahia state, Brazil (14° 28′ 27″ S; 39° 04′ 46″ W) where plants set flowers under natural conditions. This station houses a large collection of sugarcane germplasm, including relatives of this species^1^.

### Mitotic Counting and Meiotic Chromosome Behavior in IACSP93-3046

Single buds, each approximately 5 cm long, were placed in trays containing *Sphagnum* at 28°C and watered daily. Primary roots were excised and pre-treated with a 0.03% 8-hydroxyquinoline solution for 4 h at room temperature. Subsequently, the roots were fixed in 3:1 ethanol: acetic acid solution for 24 h, transferred from the fixative to a 70% ethanol solution and stored at 4°C.

The fixed roots were washed twice in distilled water, hydrolysed in 1 N HCl at 60°C for 8 min, washed again and stained according to Feulgen method (Schiff’s reagent for 45 min in the dark). After staining, they were washed thoroughly in tap water and digested in an enzyme mixture consisting of 2% cellulase (Onozuka) and 20% pectinase (Sigma) at 37°C for 1 h. The roots were then washed twice in distilled water, immersed in 45% acetic acid for 2 min, and excised root tips squashed in a drop of 1% acetic carmine. Slides were mounted in Entellan embedding agent (Merck) and examined under an Olympus BX50 microscope. Prometaphase and metaphase images were captured using an OPTIKAM B3 camera (Optika) and Optika Vision Lite 2.1 software. Twenty undamaged cells displaying well-spread chromosomes were selected for chromosome counting.

Immature inflorescences were collected and kept in a 3:1 ethanol: acetic acid solution for 24 h at room temperature. The solution was then removed and replaced with fresh fixative, and samples were stored at 4°C. Meiosis in PMCs was examined using conventional squashing procedures (Sharma and Sharma, 1980). Briefly, a flower bud was carefully dissected and anthers placed on a clean slide with a drop of 1% propionic carmine. Anthers were transversely cut off and squeezed gently to release the microsporocytes. Slides were observed under the microscope as described above. Meiotic phases were examined from metaphase I to the tetrad stage, accounting for 700 microsporocytes. Meiocytes were considered to have abnormalities if they showed irregular chromosome segregation, laggings at anaphase and telophase, chromosomes outside the nuclei at prophase II and micronuclei formation at the tetrad stage.

### Centromeric Sequence Validation for Probe Production

Genomic DNA was isolated from fresh leaves of “Caiana Fita” and IACSP93-3046 as described in Doyle and Doyle (Doyle and Doyle, 1990), with modifications in regard to the CTAB extraction buffer (2% CTAB w/v, 1.4 M NaCl, 20 mM EDTA, 100 mM Tris–HCl pH 8, 1% β-mercaptoethanol and 1% PVP 40.000 w/v) and addition of one phenol-chloroform purification step prior to the final precipitation of DNA. The quality and concentration of DNA was verified using NanoDrop 2000 (Thermo Scientific).

Next, we used information extracted from the centromeric bacterial artificial chromosome (BAC) insert of a R570 cultivar genomic library annotated in Setta et al. (2014). This BAC (SCHRBa029018; GenBank: KF184699) contains the centromeric sequence repeat (137 bp) belonging to the SCEN family described by Nagaki et al. (1998), examining an Egyptian cultivar. Our primers, CENT-F (5′-GGGTGCGTCCAAAATTATTTC-3′) and CENT-R (5′-GTACCATAGGCTCAACAATC-3′), were manually designed inside the region delimitated by the primer pair published by these authors and recognized in the BAC SCHRBa029018 sequence. The quality of the CENT primers was verified using GeneRunner v.5.0.63^2^.

Amplification reactions were carried out in a final volume of 20 μl, containing 40 ng genomic DNA of the “Caiana Fita” or IACSP93-3046 genotypes, 0.3 μM of each primer, 0.2 mM dNTP, 1.5 mM MgCl_2_, 1 × PCR buffer, and 1.0 U Go*Taq* Flexi DNA Polymerase (Promega). The cycling conditions were as follows: initial denaturation at 95°C for 5 min; 35 cycles at 95°C for 40 s, 60°C for 50 s and 72°C for 1.5 min; final extension at 72°C for 10 min. PCR products were resolved by electrophoresis in 1.2% (w/v) agarose gel stained with SYBR Safe (Invitrogen), and visualized under UV using UVP MultiDoc-It.

The PCR products were then purified (Wizard SV Gel and PCR Clean-Up System Kit, Promega) and ligated into a vector (pGEM-T Easy, Promega). Electrocompetent cells (*Escherichia coli* DH5α) were transformed and incubated in SOC medium (1 h, 37°C), and shaken at 200 rpm. The culture was spread on LB agar plates supplemented with ampicillin (50 μg/ml), X-Gal (2%), and IPTG (20%), and grown overnight (37°C). White colonies were chosen randomly and grown in liquid LB, then used to perform a plasmid DNA alkaline extraction (Sambrook and Russell, 2001). Isolated plasmids were amplified by PCR with M13 primers, allowing the insert sizes to be determined prior to submission for DNA sequencing (BigDye Terminator v3.1 Cycle Sequencing, ABI PRISM 3100 Genetic Analyzer, Applied Biosystems).

Processing to exclude low quality sequences (<100 bp, Phred < 20) and sequence assembly were carried out using CodonCode Aligner 5.1.4 (CodonCode Corporation, Dedham, MA, United States). Entire sequences (F+R) were compared with the SCEN sequence using BLASTn to identify repeats. Repeat sequences were then aligned to calculate sequence similarities in Geneious 10.1.3 (Kearse et al., 2012). The consensus sequence was obtained, and then aligned against the SCEN sequence using MUSCLE 3.8 (Edgar, 2004).

### Chromosome Association Analysis Using FISH

Anthers containing meiocytes at diakinesis were previously selected to prepare a cell suspension according to Murata and Motoyoshi (1995), with modifications. Anthers were washed in distilled water, placed in an enzyme mixture containing 2% cellulase (Onozuka), 20% pectinase (Sigma), and 1% macerozyme (Sigma) at 37°C for 10 min, and then fragmented manually with a micropipette. The suspension was centrifuged at 13,000 rpm for 5 min and the pellet washed in 50 μl of distilled water, centrifuged as above and fixed in 50 μl of an ethanol: acetic acid solution (3:1). Finally, the cells were resuspended in 30 μl of fresh fixative, and the suspension (9 μl) dropped on a clean slide dried at room temperature. Unstained slides were examined under the microscope with high contrast. The selected slides were stored at -20°C until hybridization.

FISH procedures were performed according to Schwarzacher and Heslop-Harrison (2000), with modifications. Our probes consisted of CENT repeats obtained by amplification of genomic DNA from “Caiana Fita” and IACSP93-3046, and the PCR product was purified as detailed above. Purified DNAs were labeled by nick translation (Roche) with digoxigenin-11-dUTP, following the manufacturer’s instructions.

Slides were treated with RNase (100 μg/ml, 1 h, 37°C), fixed in paraformaldehyde (4%, w/v) for 10 min, and dehydrated in an ethanol series (70% and 100% ethanol, 5 min each). The hybridization mixture consisted of formamide (50%, v/v), dextran sulfate (10%, w/v), saline sodium citrate (2 × SSC), sodium dodecyl sulfate (0.13%, w/v SDS) and 3 ng/μl of probe. A total of 15 μl of the mixture, previously denatured (10 min, 75°C), were applied to the chromosome preparations. Slides were denatured/hybridized for 10 min at each temperature (90°C, 48°C, and 38°C) using a thermocycler (5333 Mastercycler^®^, Eppendorf) and subsequently incubated in a humidity chamber (20 h, 37°C). The CENT probe was detected with anti-digoxigenin conjugated to rhodamine (Roche). Preparations were counterstained and mounted with DAPI in VECTASHIELD medium (Vector). Slides were examined under a fluorescence microscope (Olympus BX51) and images captured (Olympus DP72 camera) using DP2-BSW software (Olympus). Selected images were processed using Adobe Photoshop CS5 (Adobe Systems). Hybridization sites of the best five cells were analyzed to determine chromosome pairings.

## Results

### Mitotic Counting and Meiotic Chromosome Behavior in IACSP93-3046

**FIGURE 2:**
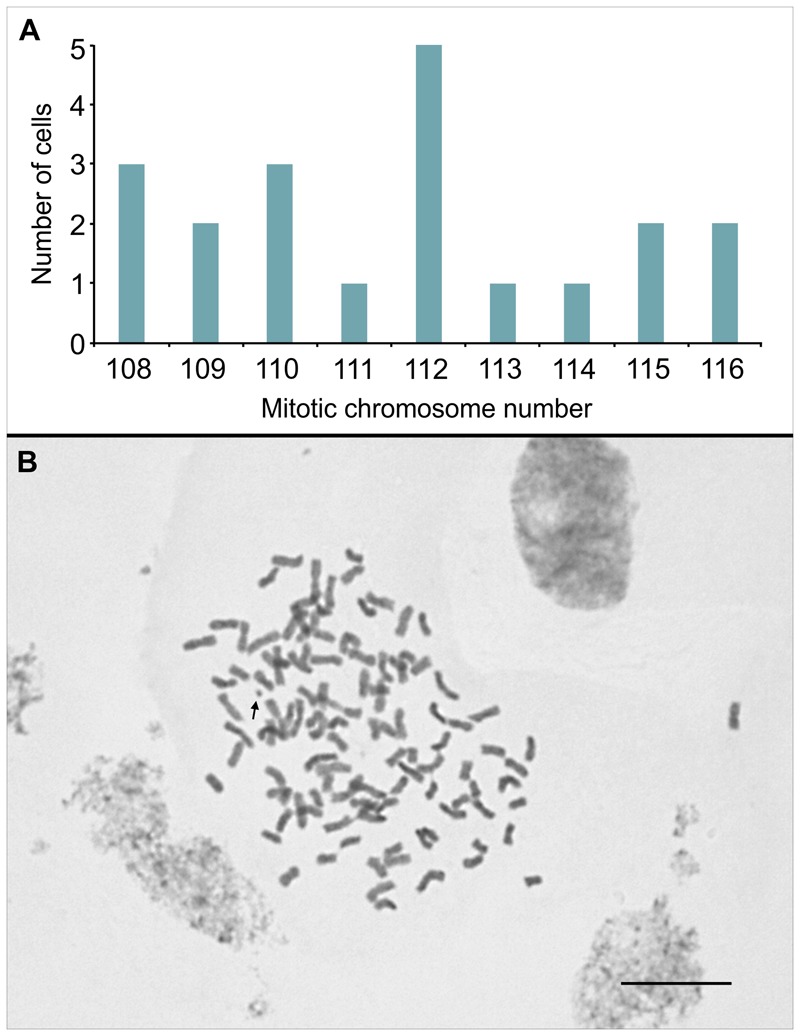
**(A)** Distribution of chromosome counts in 20 mitotic cells of IACSP93-3046, previously treated with 8-hydroxyquinoline. **(B)** A metaphase cell showing *2n* = 112 chromosomes; the arrow points to a satellite chromosome. Bar, 10 μm.

Firstly, we examined mitotic cells that were pre-treated with 8-hydroxyquinoline, a spindle-fiber inhibitor. This resulted in good chromosome spreads and accumulation of cells in prometaphase and metaphase (approx. 30 and 70%, respectively). Chromosome numbers in 20 undamaged cells varied from 108 to 116 (**Figure 2A**), and the modal value at 112 was 25%. Chromosomes were metacentric or submetacentric, with sizes ranging from 1.6 to 3.2 μm (**Figure 2B**).

A regular meiotic pattern was observed in a third of the microsporocytes (233/700), which normally underwent two divisions. The remaining cells (477/700) showed irregularities that varied in type and frequency depending on the meiotic phase (**Table 1**). For instance, we were able to recognize irregularities in 59% (145/245) and 73% (332/455) of the cells undergoing the first (excluding prophase I) and second division, respectively. We had to exclude prophase I from the meiotic analysis since only a small number of cells without superimposed chromosomes was observed, preventing us from examining chromosome pairing in conventionally stained cells, especially at diakinesis.

**Table 1:** Meiotic abnormalities observed in pollen mother cells of the IACSP93-3046 variety.

Meiotic phase	Number of cells examined	Number of cells with abnormalities^∗^	Abnormalities
Metaphase I	104	22 (21.2)	Chromosomes not lined up at the equatorial plate
Anaphase I	39	28 (71.8)	Lagging chromosomes
		4 (10.2)	Rod bivalent laggings
		7 (17.9)	Chromosome bridges
Telophase I	102	83 (81.4)	Lagging chromosomes
		5 (4.9)	Chromosome bridges
Prophase II	114	99 (86.8)	Chromosomes outside the nucleus
Metaphase II	100	57 (57.0)	Chromosomes migrating precociously to poles
		21 (21.0)	Chromosomes not aligned at the plate
Anaphase II	11	8 (72.7)	Lagging chromosomes
Telophase II	105	75 (71.4)	Laggards and chromosomes not included in the nuclei
Metaphase II/ Anaphase II	9	9 (100)	Asynchrony
Tetrad	116	63 (54.3)	Micronucleus
Total	700	477 (68.1)	

*^∗^Estimated percentage of cells with abnormalities shown in parentheses.*

**FIGURE 3:**
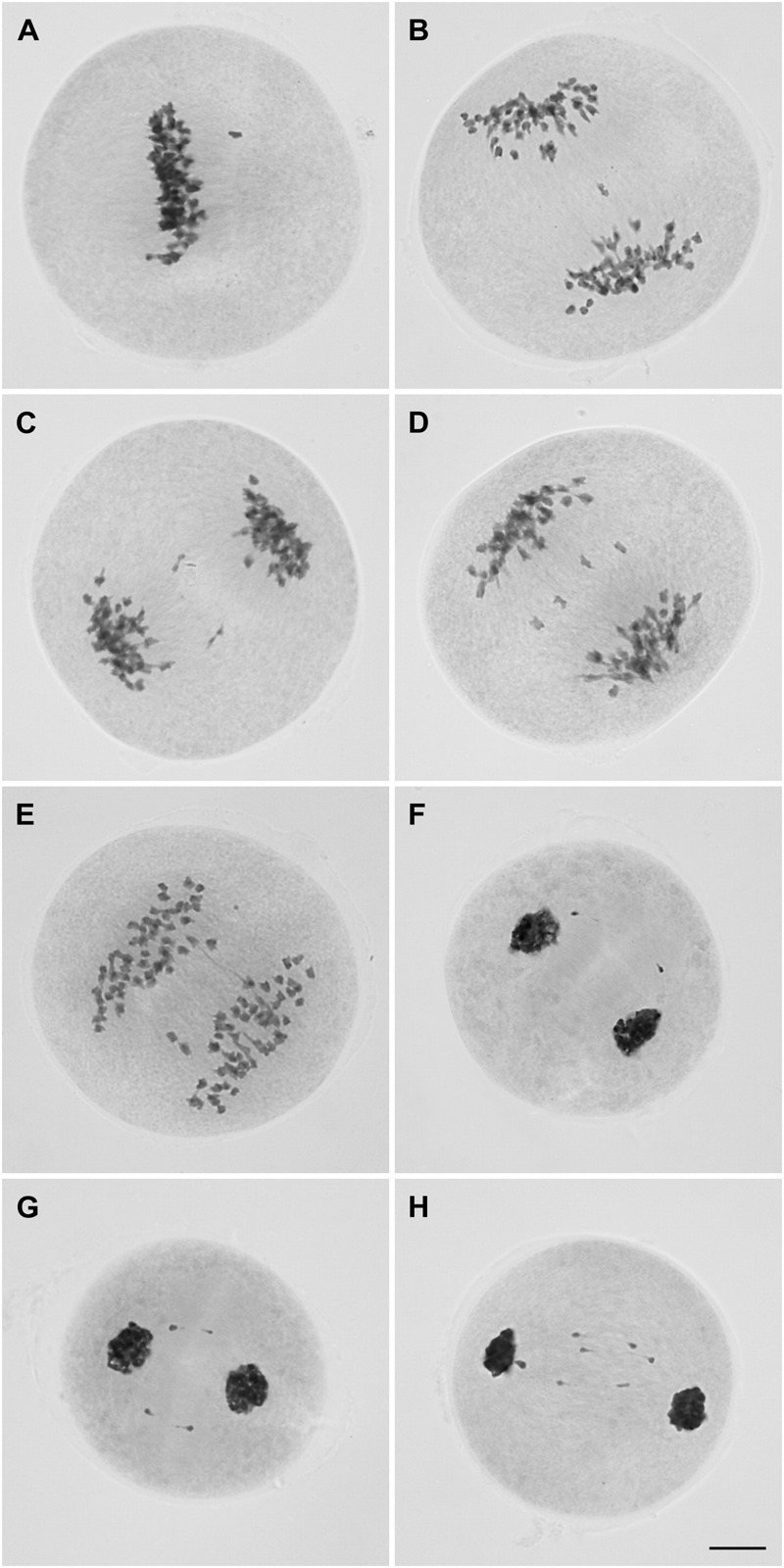
Microsporocytes of IACSP93-3046 during meiosis I showing a chromosome not lined up at the metaphase plate **(A)**, lagging chromosomes **(B-D)**, and a chromosome bridge in cells at anaphase I **(E)**. Chromosomes outside the telophase I nuclei **(F–H)**. Bar, 10 μm.

Metaphase I cells were predominantly normal, but chromosomes (1 to 2) not lined up at the equatorial plate were observed in 21% (22/104) of the cells (**Figure 3A**). From anaphase I to telophase I, the percentage of cells with irregularities was notably high (∼87%, 123/141). Cells with infrequent chromosome bridges (∼18%, 7/39) but frequent lagging chromosomes (1 to 5) were observed at anaphase I (∼72%, 28/39) (**Figures 3B–E**). Some of these laggings were rod bivalents (∼14%, 4/28) visualized in anaphase cells. One to three lagging chromosomes occurred in 86% of the anaphase cells with laggards (24/28). These figures could not be a consequence of the limited sampling of anaphase I cells (39) as such irregularities continued in telophase I, characterized by the presence of chromosomes not incorporated into new nuclei (**Figures 3F–H**). Only in some 14% (14/102) of the telophase I cells we were able to recognize two main nuclei, without lagging chromosomes. We observed 1–10 lagging chromosomes per telophase I cell; the most frequent figures were 1, 2, and 4 in approximately 13% (11/83), 29% (24/83), and 20% (17/83) of the telophase I cells with laggards, respectively.

**FIGURE 4:**
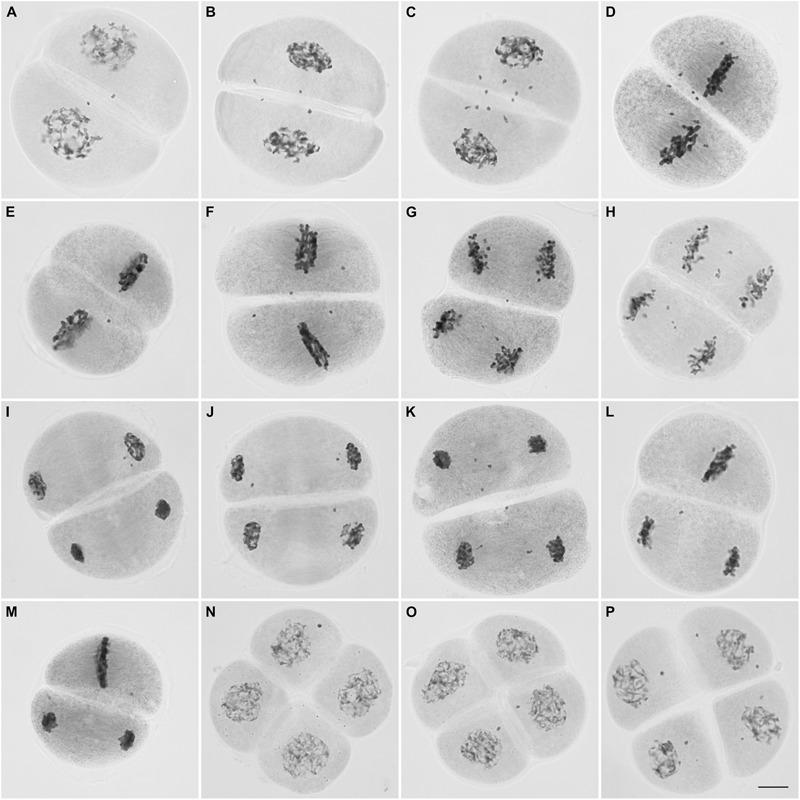
Microsporocytes of IACSP93-3046 during meiosis II. Prophase II cells showing chromosomes (2–10) not incorporated in the nuclei **(A–C)**. Chromosomes not lined up at the metaphase plate **(D–F)**. Lagging chromosomes at anaphase II **(G, H)**. Chromosomes not incorporated into the telophase II nuclei **(I–K)**. Cells showing asynchrony: one cell has completed nuclear division and the other is still at the metaphase stage **(L,M)**. Tetrad cells with micronuclei **(N–P)**. Bar, 10 μm.

During the second division, only 27% (123/455) of the cells underwent a regular pattern. Up to 10 chromosomes were observed not to be incorporated into the nuclei in ∼87% (99/114) of the prophase II cells (**Figures 4A–C**). Most of these cells (∼82%, 81/99) had one to four chromosomes not incorporated into the nuclei; the figures were: one chromosome in approximately 20% (16/81) of the cells, two chromosomes in 36% (29/81), three chromosomes in 21% (17/81) and four chromosomes in 23% (19/81).

Regular behavior was observed in 22% (22/100) of the metaphase II cells. As in prophase II, chromosomes not lined up at the plate (not attached to the spindle fibers) were visualized in 27% (21/78) of the cells or migrating precociously to the poles in the remaining cells (73%, 57/78) (**Figures 4D–F**). Lagging chromosomes were identified at anaphase II (**Figures 4G,H**), and could be those remaining and not incorporated into the telophase II nuclei (**Figures 4I–K**); the most frequent figures were 1, 2, and 4 chromosomes in 35% (26/75), 24% (18/75), and 16% (12/75), respectively. Only 29% (30/105) of the telophase II cells had complete nuclei, without micronuclei.

Interestingly, we visualized dyads with asynchronous behavior (**Figures 4L,M**); the frequency of cells showing asynchrony was 4% (9 per 225 cells at second division, excluding prophase II cells). At the end of meiosis, approximately half of the resulting daughter cells (53/116) exhibited four nuclei, but in the remaining cells (63/116) there were one to six lagging chromosomes entrapped in micronuclei. Cells exhibiting one or two micronuclei were more frequent (**Figures 4N–P**).

Summarizing, we can state that microsporogenesis in IACSP93-3046 is not regular, and chromosomal irregularities are consistent with the hypothesis of non-synchronous disjunction of bivalents (one to five) at first anaphase, resulting in lagging chromosomes (one to ten) from telophase I to subsequent phases, but visualized as entrapped in micronuclei after the second cytokinesis in the microspores. Obviously we cannot discard the possible (but infrequent) occurrence of tri- or tetravalent associations (leading to univalent segregation), although this was not observed herein, as shown below.

### Characterizing Centromeric Sequences and Fluorescent *in Situ* Hybridization With Centromeric Probes

With the aim of determining chromosome associations at prophase I, we used the FISH technique with centromeric probes. At this stage, we studied the “Caiana Fita”, a selected clone of *S. officinarum* (2*n* = 80), and the IACSP93-3046 variety. Firstly, their centromeric sequences had to be amplified, and primers were designed based on information on a centromeric sequence from a sugarcane genomic library annotated in Setta et al. (2014). As expected, amplification reactions resulted in a multiple band profile (**Supplementary Figure S1A**). After fragment cloning and insert amplification, selected clones were sequenced. Five sequences of each genotype were obtained, and 11 centromeric repeats identified in “Caiana Fita” and 12 in IACSP93-3046, and these in turn showed respective similarity of 75.7% and 72.3%. The alignment of the derived consensus sequences (with a 137 bp repeat) with that in the literature (**Supplementary Figures S1B,C**) resulted in 97.1% similarity in “Caiana Fita” and 99.3% similarity in IACSP93-3046. The GenBank accession numbers for our nucleotide sequences are: MG708493, MG708494, MG708495, MG708496 (Caiana Fita) and MG708497, MG708498, MG708499, MG708500 (IACSP96-3046).

**FIGURE 5:**
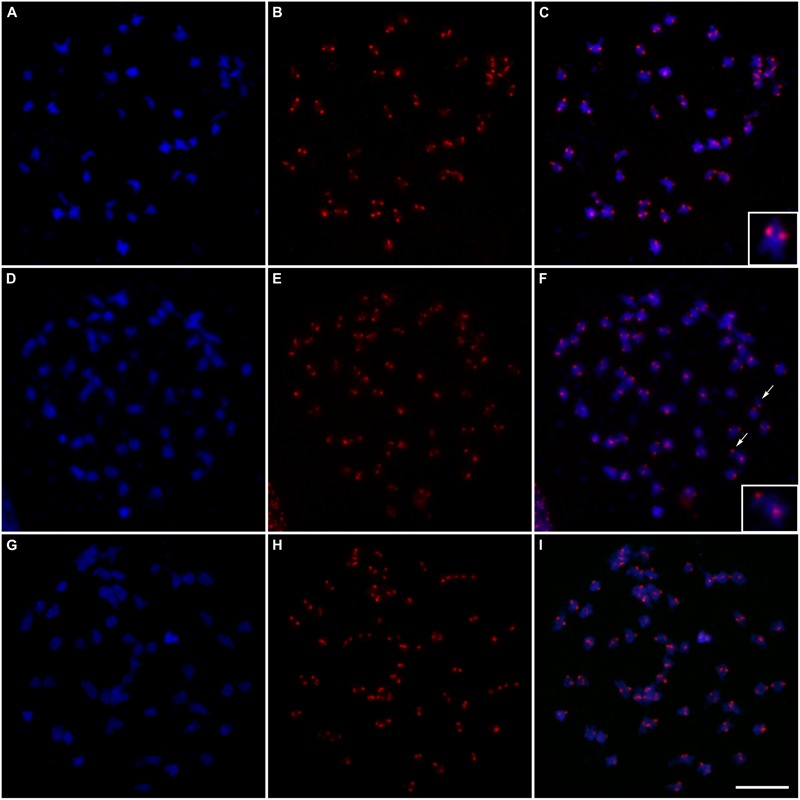
Fluorescent *in situ* hybridization of centromeric probes on meiotic cells at diakinesis: **(A)** Chromosomes of “Caiana Fita” (*Saccharum officinarum*) stained with DAPI (blue); **(B)** Centromeric sites hybridized with the CENT probe detected with anti-DIG-rhodamine (red); **(C)** Superposition of the images **(A/B)** showing 40 bivalents; the inset shows a typical bivalent; **(D,G)** Chromosomes of “IACSP93-3046 stained with DAPI (blue); **(E,H)** Centromeric sites hybridized with the CENT probe detected with anti-DIG-rhodamine (red); **(F)** Superposition of the images **(D/E)** showing 56 bivalents plus two univalents; arrows point to the univalents and the inset shows a univalent and a bivalent; **(I)** Superposition of the images **(G/H)** showing 56 bivalents. Bar, 10 μm.

Intensely fluorescent signals in the centromeric regions of “Caiana Fita” and IACSP93-3046 chromosomes were visualized at diakinesis, allowing to us to determine that bivalent associations were predominant in both genotypes (**Figures 5A–I**). Forty bivalents were usually clearly visualized at “Caiana Fita” diakinesis, although one cell exhibited 39 bivalents and 2 univalents. High prevalence of bivalent associations was also observed in the IACSP93-3046 variety (**Table 2**). In three diakinesis cells, one or two univalents were recorded. No multivalent associations were observed, but this could be a consequence of limited cell sampling.

**Table 2:** Chromosome associations observed at diakinesis of the “Caiana Fita” clone and IACSP93-3046 variety (I and II denote uni- and bivalents, respectively).

Cell	“Caiana Fita”	IACSP93-3046
1	40 II	56 II
2	40 II	56 II
3	40 II	55 II + 2 I
4	40 II	56 II + 2 I
5	39 II + 2 I	57 II + 1 I

## Discussion

Pioneering studies (Moriya, 1940; Bremer, 1961a,b) determined the chromosome numbers of POJ cultivars, including POJ2878 with *2n* = 119. In India, at the beginning of the 20th century, nobilization of *S. barberi* and *S. spontaneum* with *S. officinarum* produced early nobilized tri-species hybrids of Co seedlings (from Coimbatore), which also gained acceptance in South Africa, Australia, Louisiana (United States), Argentina and Brazil (see Paterson, 2013). Crossing POJ 2878 with Co290 produced Co419 (*2n* = 113). Both POJ2878 and Co419 are ancestors of the variety herein analyzed (**Figure 1**), which we determined to have *2n* = 112.

To clarify, the wild *S. spontaneum* (*2n* = 40–128) evolved via polyploidy and aneuploidy. It is widely distributed, a fact ascribed to its adaptability, and five major cytotypes were described with *2n* = 64, 80, 96, 112 and 128 (*x* = 8). In contrast, *S. officinarum* (*2n* = 80) is an autopolyploid, with 8 sets of chromosomes (*x* = 10); gametes have *n* = 40. Molecular evidence has confirmed the direct descent of *S. officinarum* from the wild species, *S. robustum* (*2n* = 60 to 220), and two major cytotypes were identified with *2n* = 64 and 80 (*x* = 10) (Panje and Babu, 1960; D’Hont et al., 1993; Lu et al., 1994; Amalraj and Balasundaram, 2006).

In Brazil, chromosome numbers have been determined for NA56-79 (*2n* = 114) and Co419 (*2n* = 113; Silvarolla and Aguiar-Perecin, 1994), some RB cultivars (*2n* = 110–112; Ferrari, 2010), and IAC91-1099 (*2n* = 112; Melloni et al., 2016). There are indeed some inconsistencies regarding the mitotic number and this can be ascribed to incorrect counting and the complexity of sugarcane’s origin. In fact, the majority of canes currently cultivated have *2n* = 102–119, including R570 clones (*2n* = 107–115; D’Hont et al., 1996), the variety most studied at cytological and genomic levels. The genome of R570 is composed of 80% *S. officinarum*, 10% *S. spontaneum*, and 10% recombinant chromosomes (D’Hont et al., 1996, 1998). In cultivar NCo376 (*2n* = 112), the distribution of these chromosome categories was 70:20:10%, confirming for the first time the exchanges between *S. officinarum* and *S. spontaneum* chromosomes (D’Hont, 2005). Recently, Piperidis et al. (2010) reported *2n* = 106–119 for cultivars and elite clones from Australia (5) and South Africa (2). In this elegant work, the authors used *in situ* hybridization to show that the percentage of *S. officinarum* chromosomes varied from 70% to 77%, *S. spontaneum* chromosomes 11% to 21%, and recombinants 8% to 13%, depending on the genotype.

The classic papers of Moriya (1940), Price (1957, 1963a,b, 1968), and Bremer (1961a,b) provided valuable data on the cytology of the *Saccharum* species, and studies on chromosome transmission in interspecific crosses involving *Saccharum* were undertaken following G. Bremer’s observations. The F_1_ hybrids, e.g., Kassoer, had *2n* = 136 chromosomes, equal to the sum of the maternal diploid (*S. officinarum*) and the paternal haploid (*S. spontaneum*) numbers (*2n+n* = 80+56 = 136). These F_1_ hybrids were referred to as the first nobilization of *S. spontaneum* and the subsequent backcrosses were similarly denoted. Surprisingly, regular meiosis with a large number of bivalents at diakinesis was found in this even number of chromosomes (136). Pictures of diakinesis and anaphase I shown in Bremer’s article (Bremer, 1961a) indicated 66 bivalents and four laggings near the equator of the PMCs of an interspecific F_1_ hybrid; others have shown 4–12 univalents. Later, Nair (1975) examining interspecific hybrid cells (*S. officinarum* × *S. spontaneum*) at anaphase (I and II) found lagging chromosomes and chromosome bridges, as well as micronuclei in tetrads. Remarkably, our results show similar meiotic behavior, although four generations with 8 crosses following Kassoer were performed to originate SP79-1011, the female parent of IACSP93-3046, which shows redundancy with respect to its ancestors (**Figure 1**). A question that arises is whether univalents have a pure *S. officinarum* origin or an interspecific origin (see Jannoo et al., 2004). Recombinants have arisen during the first generations due to the pairing of homeologs.

However, as widely documented for resynthesized allopolyploids (reviewed in Chen and Ni, 2006 and Grusz et al., 2017) and some natural autopolyploids such as *Arabidopsis arenosa* (Yant et al., 2013), current sugarcane cultivars are assumed to have undergone genome readjustments aimed at achieving stability and re-establishing functional pathways, including the suppression of multivalent associations by a genetic control of chromosome pairing and recombination as occurs in wheat and oat (see Naranjo and Benavente, 2015). In wheat, the locus *Ph1* ensures that recombination only occurs between homologous chromosomes. Recent experimental studies suggest that the overall effect of *Ph1* on chromosome pairing must be the promotion of homolog pairing rather than the active suppression of pairing between related chromosomes. It clearly blocks recombination or crossover between them^3^.

A reduction in chiasma number per bivalent has been suggested as a potential mechanism for meiotic diploidization in autopolyploids (as in the case of *S. officinarum*) because limiting crossovers per chromosome prevents multivalent associations (revisited in Cifuentes et al., 2010; Bomblies et al., 2016). We may speculate that pairing could be confined to homologous chromosomes or even to syndetic segments, albeit carried by recombined chromosomes. These points need to be clarified in sugarcane, and were elegantly discussed in Grandont et al. (2014) with regard to homoeologous chromosome sorting during early prophase I and progression of meiotic recombination in different *B. napus* accessions.

Burner and Legendre (1993) determined chromosome transmission and meiotic stability in sugarcane progenies derived from crosses of both elite and interspecific clones with *S. spontaneum* clones (F_1_, BC_1_, BC_2_, BC_3_). The frequency of trivalents increased linearly from one generation to the next. Pairing was primarily of bivalents, although variable numbers of univalents and multivalents were observed. Chromosome transmission was strictly *n+n*, but aneuploid gametes resulted from meiotic abnormalities, which included anaphase bridges and laggards, as well as asynchronous meiosis. Bielig et al. (2003) analyzed the frequency of *2n* microspore formation in *Saccharum* spp. hybrid clones, observing that univalents were rare and multivalents did not occur at all. The authors described the formation of dyads and triads at the end of the meiotic process in the hybrid clones, which produced *2n* gametes more frequently than did the parental species, and the production of *2n* gametes was attributed to the absence of cytokinesis rather than to a combination of asynchronous division and chromosome non-disjunction. Cytogenetic analysis confirmed that abnormal meiotic behavior existed in *Gossypium* synthetic hybrids due to non-synchronous chromosome separation at hybrid first meiosis. These abnormalities include the occurrence of a triad (partly from non-synchronized dyad separation), unequal division with abnormal unbalanced micronucleus formation, and various abnormal polyads (Wu et al., 2017).

The prevalence of unreduced gamete involvement in crosses between species and within species’ populations is suggestive of a complex set of underlying mechanisms that further support the role of unreduced gametes in facilitating polyploidy and speciation. The formation of unreduced gametes is prevalent in diverse plant species, including in *Gossypium* in which it was recently found to occur (Montes et al., 2017).

It is therefore plausible to speculate that the *Saccharum* genus has experienced a continuum between auto- and allopolyploids (Storme and Mason, 2014; Mason and Pires, 2015). Regarding *Saccharum* spp., a man-made genome, no excluding possibilities can be envisaged. Homoeologous associations allowed interspecific crossovers and the formation of recombinant chromosomes (D’Hont et al., 1996, 1998), but sugarcane adapted the mechanism to provide mostly bivalent associations during the nobilization process. It should have occurred few backcrosses later the interspecific hybridization, when the hybrid broke down to *n*+*n*. The mechanism to suppress multivalent association was possibly inherited from parental species due to the evolutionary history of polyploidy in *Saccharum*. Furthermore, our hypothesis is that PMCs of *S. officinarum* and *S. spontaneum* divide non-synchronously, and follow *Saccharum* spp. nuclei, explaining the rare formation of dyads and triads at the end of meiosis in the PMCs of current cultivars. Spindle fibers were visible in both asynchronous cells (see **Figures 4L,M**), and only tetrads were observed at the end of IACSP93-3046 meiosis.

Interspecific chromosomal exchanges have been shown to exist in the genome of modern sugarcane cultivars (D’Hont et al., 1996; Cuadrado et al., 2004; Piperidis et al., 2010), and these exchanges could be the result of homoeologous recombination or even translocation between non-homologous chromosomes. Most contemporary cultivars, including the one we analyzed, seem to be meiotically stable, since bivalent associations predominate. It is reasonable to assume that all the sugarcane chromosomes involved in preferential mutual pairing probably have a pure origin, and this applies in particular to those of *S. officinarum*. However, there is evidence that some bivalents also include chromosomal regions originating from the two ancestral species (see Jannoo et al., 2004).

None of the other currently available papers has produced high-quality meiotic preparations, as we have shown (700 meiotic cells were examined). Taking this into account and in view of the meiotic abnormalities visualized herein (**Table 1**), the following scenarios are possible. Firstly, some chromosomes undergo incomplete association leading to univalency at late prophase I. Note that our FISH results revealed the occurrence of one or two univalents at diakinesis (**Table 2**). These univalents behave as laggards in subsequent meiotic phases and may result in aneuploidy. Secondly, bi-, tri-, or tetravalent associations in early prophase may be ineffective in terms of chiasma formation and this could be part of the mechanism for inhibiting the siting of crossovers as a consequence of a gene controlled system that modifies pairing behavior in subsequent generations after the confrontation of the two genomes. Thirdly, the presence of rod bivalents (up to five visualized in late anaphase) showing the lagging tendency is consistent with a reduction in chiasma frequency (**Supplementary Figure S2A**). Finally, the presence of chromatin bridges in late anaphase I (**Supplementary Figure S2B**) is indirect evidence of the presence of paracentric inversions, although acentric chromosomes (or fragments incorporated into a micronucleus or lost) were not clearly recognized. All these mechanisms have been widely discussed in regard to natural and resynthesized polyploids, especially *Brassica* (Nicolas et al., 2007; Gaeta and Pires, 2010; Szadkowski et al., 2010).

In *S. officinarum*, univalents are very rare (Bielig et al., 2003). This applies to most established autotetraploids, in which meiotic stabilization and bivalent formation are coupled with a reduction in chiasma frequency. For example, established autotetraploids of *Arabidopsis arenosa* show a reduction in the number of crossovers per chromosome compared to the diploid ancestor (Bomblies et al., 2016). Diakinesis cells of the Thai KPS 01-01-25 sugarcane exhibited not only true chiasma bonds between homologs/homeologs, but also secondary chromosome associations resulting from loose bivalents in the absence of chiasmata (Thumjamras et al., 2016). This reduction in chiasma frequency may also indicate how sensitive the structural meiotic components are to disturbance, and could be a direct consequence of meiotic adaptation to genomic challenges (Bomblies et al., 2015).

It is worth noting that the use of centromeric probes allowed us to confirm the predominance of bivalent associations, avoiding possible errors due to the small size and high number of sugarcane chromosomes. The methodology and interpretation of results herein could provide a model for analyzing meiotic behavior in other canes, and the possible implications for breeding programs.

Furthermore, it is imperative to elucidate species-derived chromosome behavior during meiosis, and we are aware that GISH techniques combined with high quality meiotic preparations must be used if this is to be achieved. By using these techniques it will be able to trace the origins of uni- and bivalents. We also believe that chromosomal data interpretation should include genealogy information. All irregularities may vary in frequency depending on the variety, as each has its own proportion of *S. officinarum, S. spontaneum*, and recombinant chromosomes coexisting in the genome (or chromosome set). It would be interesting to discover the origins of univalents, and even bivalents, by GISH analysis, but we would have to select appropriate germplasm bank entries with usable genotypes of *S. officinarum* and *S. spontaneum*.

Currently, the global concern to seek sustainable and renewable energy has revived interest in biomass energy as an important worldwide issue (reviewed in Hoang et al., 2015). The demand for higher biomass volume to produce second generation ethanol and electricity has encouraged new directions in research for developing cultivars with higher fiber content, so-called energy cane. One strategy is to combine *Saccharum* spp. with *S. spontaneum* genome rich canes (Matsuoka et al., 2014; Hoang et al., 2015) or even *S. robustum*. It would be very interesting to analyze the consequences of this “reciprocal backcross process” in terms of the meiotic behavior of energy canes.

## Author Contributions

CA performed all cytological preparations with assistance from CO and LT under the supervision of MV and EF-M. CA, CM, and LC-S isolated and analyzed the sugarcane centromeric sequences under the supervision of CM-V. LP and MX provided the plant material and information. MV conceived the study and wrote the article.

## Conflict of Interest Statement

The authors declare that the research was conducted in the absence of any commercial or financial relationships that could be construed as a potential conflict of interest.

## Footnotes

1 http://www.iac.sp.gov.br/areasdepesquisa/cana/index.php

2 http://www.generunner.net/

3 https://www.jic.ac.uk/staff/graham-moore/Wheat_meiosis.htm

## Supplementary Material

FIGURE S1 **(A)** Agarose electrophoresis of PCR products amplified by the primer pair, CENT-F plus CENT-R. Genomic DNAs used as templates: “Caiana Fita” (lane 1) and IACSP93-3046 (lane 2); C, control reaction, without template DNA; M, 100 bp DNA ladder (Invitrogen). **(B,C)** Alignment of the centromeric consensus sequences of “Caiana Fita” (Repeat 1) and IACSP93-3046 (Repeat 2) against the SCEN repeat (Nagaki et al., 1998).

FIGURE S2 Anaphase cells showing a rod bivalent lagging chromosome (**A**, arrowed) and chromosomal bridges **(B)**.
