# Corrigendum: Revisiting Meiosis in Sugarcane: Chromosomal Irregularities and the Prevalence of Bivalent Configurations

**DOI:** 10.3389/fgene.2019.00287

**Published:** 2019-04-03

**Authors:** Maria Lucia C. Vieira, Carmelice B. Almeida, Carlos A. Oliveira, Luana O. Tacuatiá, Carla F. Munhoz, Luiz A. Cauz-Santos, Luciana R. Pinto, Claudia B. Monteiro-Vitorello, Mauro A. Xavier, Eliana R. Forni-Martins

**Affiliations:** ^1^Escola Superior de Agricultura “Luiz de Queiroz”, Universidade de São Paulo, Piracicaba, Brazil; ^2^Instituto de Biologia, Universidade Estadual de Campinas, Campinas, Brazil; ^3^Centro de Cana, Instituto Agronômico de Campinas, Ribeirão Preto, Brazil

**Keywords:** *Saccharum* spp., meiotic behavior, meiotic irregularities, FISH, centromeric probes, chromosome associations

There is an error in the Funding statement. The correct number for Fundação de Amparo a Pesquisa do Estado de São Paulo (FAPESP) is “2008/03969-4.”

The authors apologize for this error and state that this does not change the scientific conclusions of the article in any way. The original article has been updated.

