# Supplementary figures and images for: Revisiting Meiosis in Sugarcane: Chromosomal Irregularities and the Prevalence of Bivalent Configurations
Source: Front Genet. 2018 Jun 14;9:213. doi: 10.3389/fgene.2018.00213 (PMC6010537; doi:10.3389/fgene.2018.00213)

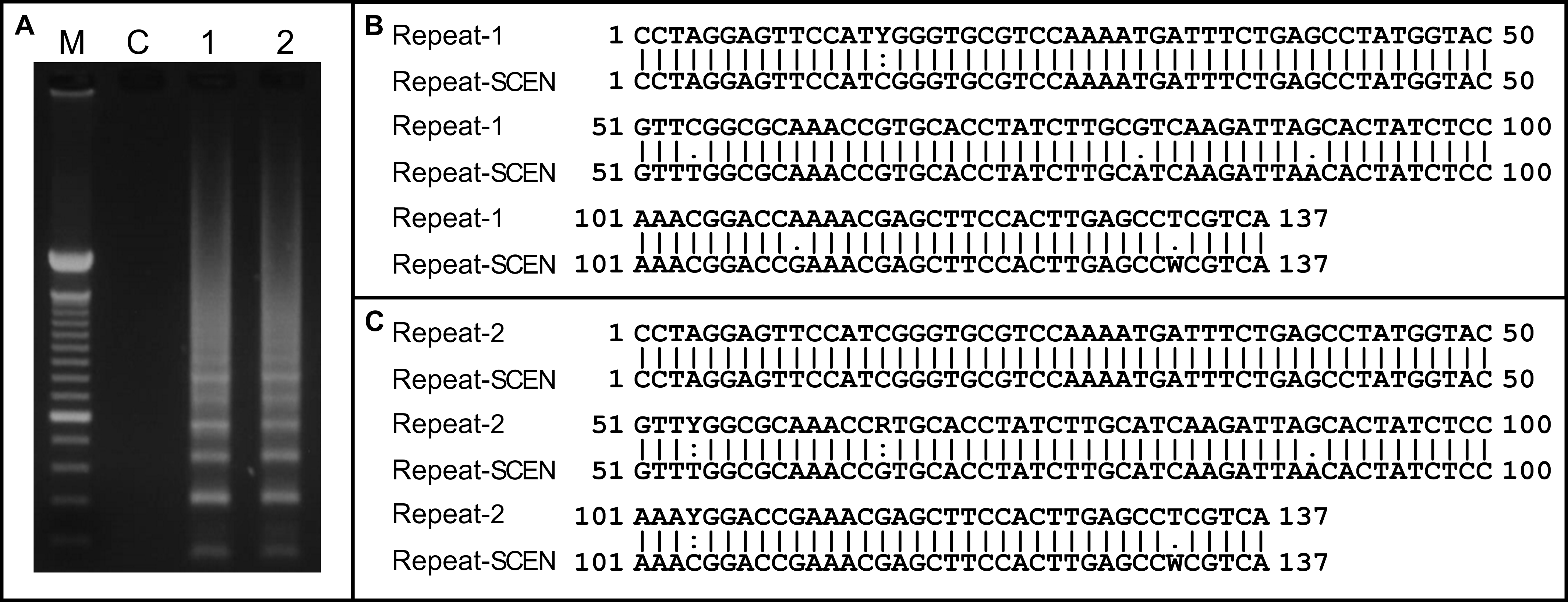

Supplement: FIGURE S1 — (A) Agarose electrophoresis of PCR products amplified by the primer pair, CENT-F plus CENT-R. Genomic DNAs used as templates: “Caiana Fita” (lane 1) and IACSP93-3046 (lane 2); C, control reaction, without template DNA; M, 100 bp DNA ladder (Invitrogen). (B,C) Alignment of the centromeric consensus sequences of “Caiana Fita” (Repeat 1) and IACSP93-3046 (Repeat 2) against the SCEN repeat (Nagaki et al., 1998). [file Image_1.TIF]

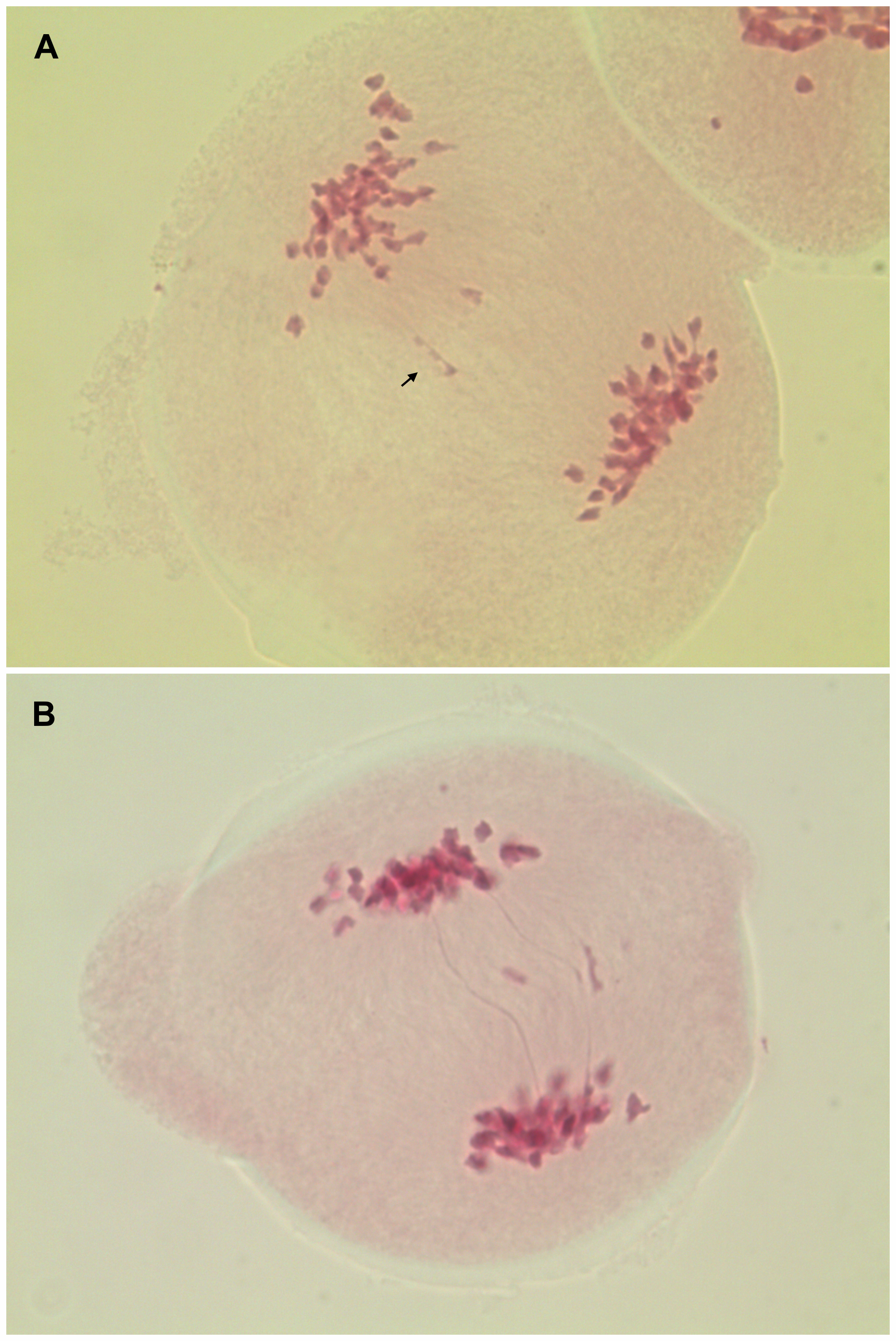

Supplement: FIGURE S2 — Anaphase cells showing a rod bivalent lagging chromosome (A, arrowed) and chromosomal bridges (B). [file Image_2.TIF]
